# Early birds and night owls: natural variation of circadian traits in plants

**DOI:** 10.1093/jxb/erag013

**Published:** 2026-01-14

**Authors:** Ann Feke, Eva M Farré

**Affiliations:** Department of Plant Biology, Michigan State University, East Lansing, MI 48824, USA; Department of Biology & Earth Science, Otterbein University, Westerville, OH 43081, USA; Department of Plant Biology, Michigan State University, East Lansing, MI 48824, USA; Max-Planck-Institut fur Molekulare Pflanzenphysiologie, Germany

**Keywords:** Adaptation, circadian clock, circadian period, flowering, natural variation, photoperiod

## Abstract

Circadian clocks have long been hypothesized to tightly link cellular and physiological processes to the appropriate time within the 24-hour cycle of the Earth’s daily rotation. According to this hypothesis, circadian rhythms with cycle lengths that differ significantly from 24 hours would be disadvantageous, as they would generate a desynchronization between the endogenous and exogenous cycles that would place stress upon an organism through the required daily resetting at dawn. However, recent work has demonstrated that endogenous circadian cycles that differ from 24 hours by 2 hours or more are prevalent within the green lineage. Herein, we review recent work on the prevalence of, and adaptive advantages associated with, natural variation in circadian cycles. Based on known photoperiodic sensing mechanisms we also describe a set of principles that allow the same changes in circadian period to cause different plant responses. This fine-tuning of clock output pathways provides a flexible mechanism enabling plants to use a wide range of life history strategies for plant adaptation to different environmental niches. Further studies are needed to determine how variations of the clock and other signals are integrated in different plants. These studies highlight the circadian clocks’ position as a prime adaptation target for migration of plant species into new environmental ranges.

## Teaser

This review summarizes the current evidence for an adaptive role of the circadian clock in wild plants. We explore the hypothesis that variation in clock output pathways enables circadian traits to lead to different phenotypic responses, permitting plants the use of different life history strategies to optimize growth and survival.

## Introduction

**Fig. 1.**
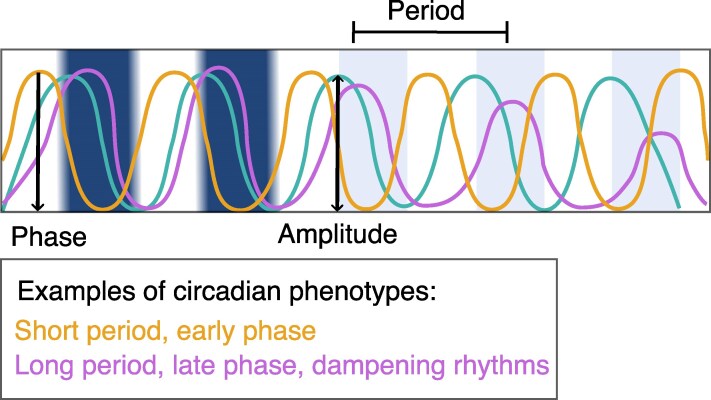
Characteristics of diel and circadian rhythms. Circadian rhythms are maintained under constant environmental conditions. The period length is the length of one rhythmic cycle, period lengths are 24 hours under light/dark conditions but vary based on the circadian control under constant environmental conditions. Phase is the time, relative to one cycle, that a rhythmic response occurs. Amplitude is the magnitude of the oscillation; it decreases when rhythms dampen. The period, phase, and amplitude can vary among genotypes.

As organisms living on Earth’s surface, plants need to adjust to predictable rhythmic changes in environmental conditions that occur on daily or yearly scales. Circadian clocks enable plants to tell the time of day. In addition, plants use this capacity to tell time in conjunction with light signals to monitor daylength changes and detect the seasons occurring at higher latitudes. Circadian clocks are molecular oscillators that are able to maintain rhythms in the absence of environmental cues, also known as free-running conditions. The Earth’s rotation time is currently very close to 24 hours (23 hours, 56 minutes, and 4 seconds) although has varied by ∼6 hours in the past 2 billion years (Mitchell and Kirscher, 2023). The clock’s ‘free running’ oscillations have a periodicity of about 24 hours when measured in the laboratory, therefore the name ‘circa-dian’. [Circadian comes from the Latin, combining *circa* (about) and *dies* (day).] However, the characteristics of these rhythms (period, or the duration of the cycle; amplitude, or the amount of change within a single cycle; robustness, or the ability of the cycle to maintain precision and/or amplitude over time; and sensitivity to environmental factors) can vary considerably across genotypes (Fig. 1, Table 1).

**Table 1.** Variation of circadian traits across wild plant populations. Summary of studies describing natural variation of circadian traits in wild species. The range of the period length in wild populations was estimated based on the published data. The Pearson correlation r^2^ values are given for statistically significant correlations as reported by the authors. When a r value was published, the r^2^ was calculated to aid comparisons across studies

Species	Wild plant source	Method	Circadian phenotype displaying natural variation	References	Correlation with environmental or geographic parameters	Other phenology
*Arabidopsis thaliana*	Accessions	Leaf movement	Period (∼22–29 h)	(Michael *et al.*, 2003b)	Period versus daylength (r^2^=0.08)	
*Arabidopsis thaliana*	Accessions	Leaf movement	Period (∼23–27 h) and temperature compensation	(Edwards *et al.*, 2005)	Period versus elevation (r^2^=0.19–0.33)	
*Arabidopsis thaliana*	Accessions	Stable bioluminescence reporter, leaf movement	Period (∼20–25 h), robustness and temperature compensation	(Kusakina *et al.*, 2014)	Temperature compensation versus growth (r^2^=0.12)	Less temperature compensation leads to more dry weight gain at high temperature (r^2^=0.22–0.33)
*Arabidopsis thaliana*	Accessions	Stable bioluminescence reporter	Phase, period	(de Montaigu *et al.*, 2015; De Montaigu and Coupland, 2017)		Later *GI* phase leads to longer hypocotyl length (r^2^=∼0.36)
*Arabidopsis thaliana*	Accessions	Delayed fluorescence	Period (∼21–26 h), phase, robustness	(Rees *et al.*, 2021)	Period versus latitude (r^2^=0.06), period versus longitude (r^2^=0.09)	Flowering time versus (r^2^=0.28), freezing tolerance, seed dormancy
*Brassica rapa*	Wild population	Leaf movement	Period (∼25–27 h), phase, amplitude, temperature compensation	(Lou *et al.*, 2011)		
*Boechera stricta*	Wild populations and families	Leaf movement	Period (∼21–24 h)	(Salmela *et al.*, 2016)		Period versus growth (r^2^=0.43)
*Boechera stricta*	Families within one population	Leaf movement	Period (∼21–24 h)	(Salmela *et al.*, 2018)		Flowering time (r^2^=−0.38), midday leaf starch (r^2^=−0.76)
*Boechera stricta*	Wild populations and families	Leaf movement	Period (∼20–30 h)	(McMinn *et al.*, 2022)	Elevation versus period (r^2^=0.23), elevation versus period range (r^2^=0.35)	
*Hordeum vulgare*	Accessions	Chlorophyll fluorescence traits	Period (24–27 h), rhythm robustness, amplitude	(Dakhiya *et al.*, 2017)	Amplitude versus organic matter (r^2^=0.42)	
*Lemna aequinoctialis*	Accessions	Transient bioluminescence reporter	Period (∼20–28 h)	(Muranaka *et al.*, 2022)		Period versus critical daylength (r^2^=0.37)
*Lemnaceae*	Species/accessions	Transient bioluminescence reporter	Period (24–36 h), temperature compensation	(Isoda *et al.*, 2022)	Latitude versus rhythm robustness (qualitative evaluation)	
*Mimulus guttatus*	Populations	Leaf movement	Period (∼22–27 h)	(Greenham *et al.*, 2017)	Latitude versus period (annual populations, r^2^=0.36)	
*Mimulus laciniatus*	Populations	Leaf movement	Period (24–26 h)	(Leinonen *et al.*, 2020)	Elevation	Flowering, seed ripening
*Solanaceae* (potato)	Accessions	Leaf movement	Period (∼21–26 h), phase	(Müller *et al.*, 2016)		
*Solanaceae* (potato)	Species	Delayed fluorescence	Period (19–27 h)	(Hardigan *et al.*, 2017)	Latitude (qualitative)	

Natural selection works towards ensuring that physiological processes occur at the most beneficial time of the day. Under natural conditions, the circadian clock is entrained by the environmental inputs, or *zeitgebers*, such as light and temperature, which cycle throughout the day. Therefore, the period under driven conditions, wherein a plant is given *zeitgeber* signals, is always 24 hours. The clock sets this timing based on the proportion of the cycle at which the process should occur. Thus, although dawn, the light *zeitgeber*, is able to reset the clock every 24 hours, the innate pacing of the circadian clock will determine the number of hours after dawn that a process occurs.

Variation in circadian clock-associated genes have been linked to the domestication and improvement of numerous plant crops, and these results have been summarized in excellent recent reviews (Dwivedi *et al.*, 2024; Gerhardt and Mehta, 2025; González-Delgado *et al.*, 2025b). However, only a few of these variants of clock gene orthologues have been demonstrated to influence circadian traits. Therefore, the goal of this paper is to focus on the variation of circadian traits across wild plants and discuss their potential role in local adaptation to natural environments.

We first provide a brief overview of the mechanisms regulating the clock in the model plant *Arabidopsis thaliana*, since that is the primary framework currently used to understand how genetic variation leads to changes in rhythms and other physiological processes. Furthermore, the work performed on multiple species has shown that the genes and mechanisms involved in generating clock function are largely conserved across land plants (Linde *et al.*, 2017; Petersen *et al.*, 2022). We then describe how circadian traits are measured and discuss factors to consider when carrying out and interpreting these studies using natural populations. Finally, we summarize the current status of variation of circadian traits across natural populations and their links to potential local adaptation. Although this review focuses on wild plants, we introduce discoveries made in crops where helpful to provide potential mechanisms of regulation under natural environments.

### Key genes involved in circadian control in plants

**Fig. 2.**
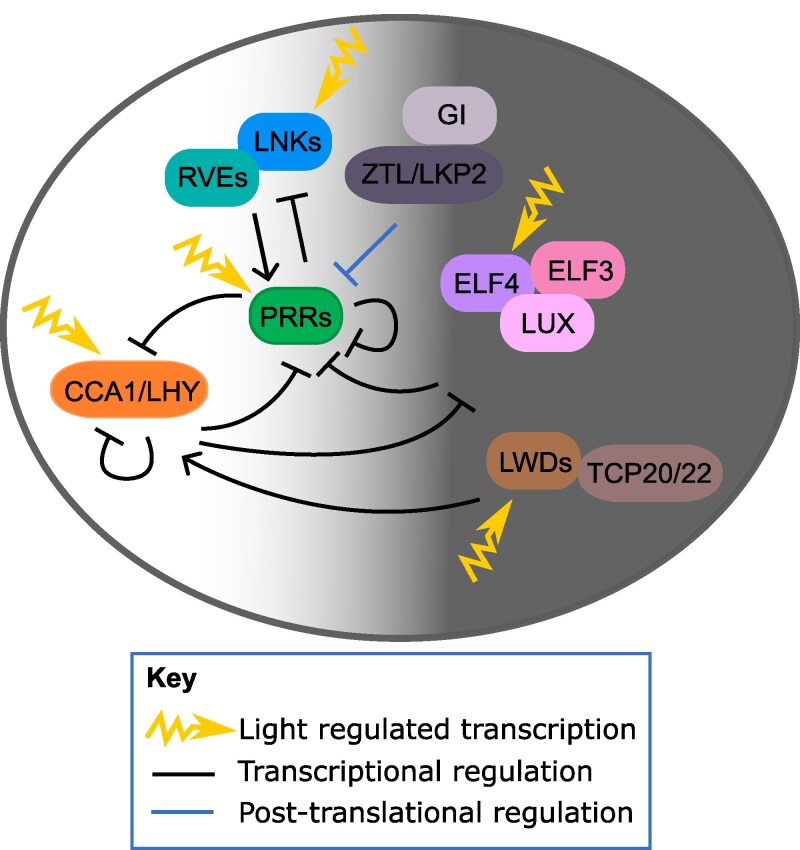
Abbreviated representation of the *Arabidopsis thaliana* circadian regulatory network. Arrows represent activation and blunt ends, repression. The light area represents the day and the dark area the night. The clock components are arranged by the time of expression throughout the day/night cycle. CCA1 (CIRCADIAN CLOCK ASSOCIATED 1), LHY (LATE ELONGATED HYPOCOTYL), RVE (REVEILLE), LNK (NIGHT LIGHT-INDUCIBLE AND CLOCK-REGULATED), PRR (PSEUDO-RESPONSE REGULATOR), GI (GIGANTEA), ZTL (ZEITLUPE), LKP2 (LOV KELCH PROTEIN 2), ELF4 (EARLY FLOWERING 4), ELF3 (EARLY FLOWERING 3), LUX (LUX ARRHYTHMO), LWD (LIGHT-REGULATED WD), TCP (TEOSINTE BRANCHED 1, CYCLOIDEA, and PROLIFERATING CELL FACTOR).

Circadian clocks are largely built of cyclic feedback loops of transcription factors that regulate one another’s expression (Fig. 2). This regulatory network is capable of maintaining rhythms in the absence of external stimuli across a wide range of physiological temperatures, a characteristic named temperature compensation. In plants, the bulk of the work on the function of the circadian clock has been performed in the angiosperm *Arabidopsis thaliana*, although work in a wide range of species indicates conservation of clock components across the green lineage (Petersen *et al.*, 2022).

In Arabidopsis, this network of feedback loops predominantly consists of three groups of repressors (Harmer, 2025). The first group of repressors consists of the SHAQY MYB domain transcription factors *CIRCADIAN CLOCK ASSOCIATED 1* (*CCA1*) and *LATE ELONGATED HYPOCOTYL* (*LHY*), two homologous MYB-type transcription factors that are expressed near dawn. These genes are repressed by the *PSEUDO-RESPONSE REGULATORs* (*PRR1/5/7/9*), which are expressed sequentially in the late afternoon into the early evening, starting with *PRR9* and ending with *PRR1* (also known as *TIMING OF CAB EXPRESSION 1* or *TOC1*). An additional, unrelated transcription factor, the TEOSINTE BRANCHED1-CYCLOIDEA-PCF (TCP) transcription factor CCA1 HIKING EXPEDITION (CHE), physically interacts with TOC1 in order to assist in the repression of the dawn-phased MYBs (Pruneda-Paz *et al.*, 2009). As *CCA1* and *LHY* expression is reduced during the day, this releases the repression on the evening-phased *EARLY FLOWERING 3* (*ELF3*), *ELF4*, and *LUX ARRHYTHMO* (*LUX*, also known as *PHYTOCLOCK 1* or *PCL1*) and its homologue *NOX* (also known as *BROTHER OF LUX*, or *BOA*) (Dai *et al.*, 2011; Nusinow *et al.*, 2011). LUX has been shown to have a traditional DNA binding domain, and it is part of a protein complex consisting of all three evening-phased genes, known as the Evening Complex (EC), which acts as a repressor of several *PRRs* (Silva *et al.*, 2020). Reduction in *PRR* expression leads to a subsequent release on the repression of *CCA1* and *LHY* during the night (Nakamichi *et al.*, 2010).

In addition to these three groups of repressors, several groups of transcriptional activators have also been identified in clock function. The REVEILLEs (RVEs) are a group of MYB-type transcription factors closely related to CCA1 and LHY which, through physical association with the NIGHT LIGHT-INDUCIBLE AND CLOCK-REGULATED (LNK) proteins, act as transcriptional activators of the *PRR* and Evening Complex genes (Rawat *et al.*, 2011; Hsu *et al.*, 2013; Hughes and Harmer, 2023; Kidokoro *et al.*, 2023). A second group of transcriptional activators, the LIGHT-REGULATED WDs (LWDs) activate the expression of both *CCA1*/*LHY* and the *PRR*s. Two TCP transcription factors, TCP20 and TCP22, are also believed to act in conjunction with the LWDs to activate the expression of morning-phased clock genes (Wu *et al.*, 2016).

Outside of this complex transcriptional network, post-transcriptional regulation has also been shown to play a role in clock tuning. For example, alternative splicing of *CCA1* that regulates the amount of fully functional protein produced plays a significant role in temperature compensation, or the circadian clock’s ability to maintain pacing throughout varying environmental temperatures (Seo *et al.*, 2012). Photoreceptors such as the F-box type E3 ubiquitin ligase ZEITLUPE (ZTL) and its homologue LOV KELCH PROTEIN 2 (LKP2), the PHYTOCHROMES (PHY), and CRYPTOCHROMES mediate light input to the clock (Oakenfull and Davis, 2017). EMPFINDLICHER IM DUNKELROTEN LICHT 1 (EID1) is involved in PHYA signalling in Arabidopsis, and has been demonstrated to be involved in clock regulation in soybean and tomato (Marrocco *et al.*, 2006; Müller *et al.*, 2016; Qin *et al.*, 2023). Additionally, GIGANTEA (GI) is believed to be a core component of the circadian clock, at least in part by regulating the ability of ZTL to degrade its target proteins, TOC1, PRR5, and CHE, as well as modulating chromatin accessibility (Cha *et al.*, 2017; Lee *et al.*, 2018).

The loss of any clock component, in the absence of genetic redundancy, leads to changes in the circadian rhythms, such as period, amplitude, and/or phase (Harmer, 2025). In addition, Arabidopsis clock components have been shown to directly influence the expression of multiple other genes and directly modulate a wide range of physiological responses such as growth, photoperiod sensing, and biotic and abiotic stresses. These studies have been recently reviewed (Xu *et al.*, 2022; Li *et al.*, 2025). Mechanistic studies in Arabidopsis have shown how circadian clock components act as coordinators of physiological responses in plants by integrating numerous environmental and endogenous signals. For example, *PRR7* transcription is regulated by sugars (Frank *et al.*, 2018) and its protein level is controlled in a light dependent manner (Farré and Kay, 2007). In turn, PRR7 not only acts as a transcriptional regulator of a large number of genes (Liu *et al.*, 2013) but also modulates the protein stability of the photoperiod control protein CONSTANS (CO) (Hayama *et al.*, 2017). In accordance, allelic variation of clock genes in Arabidopsis accessions leads to variation in circadian traits and clock-controlled responses. For example, an *ELF3* variant found in the Arabidopsis Shahdara accession caused a short period in constant light, a weaker shade avoidance response, and early flowering (Jiménez-Gómez *et al.*, 2010; Coluccio *et al.*, 2011; Anwer *et al.*, 2014).

The core characteristics of the plant circadian clock, such as the involvement of pseudo-response regulators and SHAQY MYB domain transcription factors appear to be largely conserved within the green lineage (Linde *et al.*, 2017; Petersen *et al.*, 2022). However, the complexity and genetic redundancy appears to have increased during plant evolution. Lineage-specific copy number variation and neofunctionalization occurs across plant species (Lou *et al.*, 2011; Calixto *et al.*, 2015; Linde *et al.*, 2017). Although in most cases studied orthologues of clock-associated genes continue to be regulated by the circadian clock themselves, some retain a clock function but others appear to lose it. For example, numerous variants of Arabidopsis clock gene orthologues have been associated with photoperiod insensitivity in crops (González-Delgado *et al.*, 2025b). It is interesting that most of these variants are localized in a relatively small number of genes, in particular *ELF3*, *PRR3/7*, and *LUX* across different species (Gerhardt and Mehta, 2025; González-Delgado *et al.*, 2025b). Work on various crop species indicates that the effects on photoperiod sensing caused by clock orthologue variants are not always linked to changes in circadian traits (Turner *et al.*, 2005; Faure *et al.*, 2012; Gao *et al.*, 2014; Wittern *et al.*, 2023; Helmsorig *et al.*, 2024), suggesting that either these genes have specialized into photoperiod response genes or redundancy covers their clock function. However, clock trait changes in other variants support the notion that although neofunctionalization occurs within clock-associated gene families, the core clock regulatory network contains similar components across different plant species. Work on Arabidopsis and crop species supports the strong pleiotropy of clock-associated genes, which has been linked to the adaptive role for the circadian clock (Oravec and Greenham, 2022; González-Delgado *et al.*, 2025b).

### Role of the circadian clock on adaptation to 24-hour cycles

Initial attempts to investigate the role of the clock on fitness and adaptation investigated the effects of daily cycles of either shorter or longer durations, such as 20 or 28 hours, while maintaining the same ratio of light and dark periods. In cyanobacteria, mutants with an endogenous circadian period close to the environmental cycle length outperform mutants in which these periods do not match (Woelfle *et al.*, 2004). Similar effects of matching endogenous and environmental periods have been observed in Arabidopsis mutants. In one of these studies, the fitness of an F_2_ population of Arabidopsis from a cross between *prr7prr9* (long period) and *prr5prr7* (short period/arrhythmic) was tested under either 20- or 28-hour cycles (T-cycles) (Yerushalmi *et al.*, 2011). When the F_2_ was grown under 28-hour cycles, a higher proportion of F_3_ plants had a longer period. The opposite was true under 20-hour T-cycle selection, supporting the notion that a match between endogenous and environmental cycle length improves fitness and survival. In this case, the optimization of biomass production, the timing of flowering, or a combination of the two could be responsible for these differences on fitness, since *PRR* genes have been shown to influence both responses (Nakamichi *et al.*, 2007; Ruts *et al.*, 2012; Hayama *et al.*, 2017). However, it is also important to highlight the observation that when assessing biomass of long- and short-period Arabidopsis mutants, both types of mutants still display maximum biomass under 24-hour cycles rather than under either shorter or longer T-cycles (Graf *et al.*, 2010). These observations indicate that factors other than the endogenous period affect growth in circadian mutants. Similar experiments, however, have not yet been conducted on plants that naturally have different period lengths. These experiments under T-cycles that differ from 24 hours help us address the reason of why clocks originated initially, but they are unlikely to explain the strong diversity of circadian traits we observe in wild plant populations. For example, work on the Arabidopsis Col-0 accession demonstrated that dawn and dusk are not anticipated well in environmental cycles that strongly differ from its endogenous period of ∼24 hours (Dodd *et al.*, 2014). However, the endogenous period in natural populations can vary from about ∼18 to 30 hours and therefore strongly differs from the natural 24-hour environmental cycle (Table 1) raising the question of what is the role of this variation in plant fitness and adaptation under natural conditions.

### Factors to consider when measuring natural variation of circadian traits

To understand the role of the circadian clock on adaptation it is necessary to monitor its traits across a wide range of genotypes. Any physiological response that displays free-running circadian rhythms can be used to monitor clock traits, such as phase, period, amplitude, and temperature compensation, which are measured under constant environmental conditions. High time resolution and multiple days of data are required for accurate quantification of these traits. There are several circadian-regulated responses that are conducive to high-throughput monitoring of a large number of plants and have been used to monitor natural variation of the circadian clock (Table 2).

**Table 2.** Non-invasive methods for high throughput monitoring of circadian rhythms in plants

Method	Pros	Cons	References
Leaf movement	Low-cost set-up Genetic transformation not required Relies on growth	Image analysis can be challenging Grasses have no circadian growth rhythms Data can be noisy Affected by temperature Needs infrared light source to monitor rhythms under light/dark Relies on growth	(Müller and Jiménez-Gómez, 2016; Wagner *et al.*, 2017; Lou *et al*., 2022)
Chlorophyll fluorescence —delayed fluorescence	Genetic transformation not required Works with grasses and algae	Data can be noisy Elevated cost of camera system Can only be used under constant light	(Goltsev *et al.*, 2009; Rees *et al.*, 2019)
Chlorophyll fluorescence—photosynthetic parameters	Genetic transformation not required Works with grasses and algae	Data can be noisy Complex light regimes required Elevated cost of camera system Can only be used under constant light	(Litthauer *et al.*, 2015; Cano-Ramirez *et al.*, 2018)
Transcriptional bioluminescence reporters	Robust rhythms Depending on the reporter, it can be used in all environmental conditions	Elevated cost of camera system Requires genetic transformation	(Muranaka *et al.*, 2015; Sorkin *et al.*, 2023)
Thermal imaging	Genetic transformation not required Functions in grasses	Potential elevated cost of camera system Can only be used under constant light	(Dakhiya and Green, 2019; Dong *et al.*, 2024)

Environmental conditions, such as light (quality and quantity), temperature, nutrients, carbon and water availability, the microbiome, and biotic and abiotic stresses can influence free-running oscillations (Zhang *et al.*, 2023; Dorling *et al.*, 2025). Therefore, these factors need to be taken into consideration when interpreting circadian phenotyping experiments. These phenomena occur because clock components, as mentioned above, are not isolated from other parts of the cell signalling network. The constant environmental conditions necessary to quantify circadian traits are necessarily artificial and need to be considered as a ‘common or garden experiment’ when comparing multiple natural variants. Moreover, mimicking natural growth conditions with respect to light and temperature for free-running experiments is not always feasible. For example, some plants are damaged by constant light at higher quantities and some temperatures might not allow sufficient growth to allow the quantification of rhythmic leaf movements which rely on elongation. High temperatures have also been associated with lower robustness and higher variability of rhythms in different species and different reporters (Kusakina *et al.*, 2014; Bdolach *et al.*, 2019; Prusty *et al.*, 2021; Isoda *et al.*, 2022).

Plants need to be entrained (synchronized) before circadian monitoring in constant conditions. These entrainment conditions, such as photoperiod length or temperature, have also been shown to influence free-running rhythm traits (Dodd *et al.*, 2014; Rubin *et al.*, 2017). However, this work in Arabidopsis has demonstrated that the phase of entrainment is affected most by different light entrainment regimes, in contrast to the free-running period, which remains more robust when stable rhythmicity is reached after transfer to constant conditions. In domesticated rice, detailed analysis of rhythmic gene expression in the field indicates that although gene expression is modulated by light and, in particular, temperature, it retains the capacity of maintaining the internal time in spite of fluctuations of the outside environment (Matsuzaki *et al.*, 2015). These observations might explain why the circadian period, rather than phase or amplitude, better reflect the internal time measurement mechanism in plants.

It is also important to be aware that each circadian-regulated response reflects the rhythms of the cells and tissues involved in that response. For example, differences in temperature sensitivity have been observed between bioluminescence reporters that show cell-specific expression (Michael *et al.*, 2003a) and although there is co-ordination across cells and tissues in plants, differences in rhythms have been observed across organs, tissues, and cell types (Greenwood *et al.*, 2022; Mortada *et al.*, 2024; Qin *et al.*, 2025; Zulfiqar *et al.*, 2025, Preprint). Finally, when comparing results across studies it is also important to take into consideration the developmental stage of the plants since changes in rhythmicity have been observed in plants of different ages (Kim *et al.*, 2016) and rhythms have been shown to be disrupted in endodormant trees (Ramos *et al.*, 2005).

In summary, it is necessary to critically evaluate differences between phenotypes that might be due to different experimental conditions, the type of reporter or developmental stage of the plant studied. For example, differences in the temperature-dependent changes of the circadian period between leaf movement and bioluminescence reporters have been reported in Arabidopsis accessions (Kusakina *et al.*, 2014). Both phenotypes were measured under different light qualities and quantities. These results might suggest that light signals influence temperature compensation as has been previously suggested (Pooam *et al.*, 2021). Finally, the effect of temperature on amplitude changes can vary depending on the output measured (Prusty *et al.*, 2021; Estravis-Barcala *et al.*, 2025). In spite of all these potential caveats, as we discuss below, measurements in laboratory free-running conditions have been shown to correlate with other phenotypes measured under more natural conditions, indicating that these circadian traits might be relevant to understanding the role of the plant clock in the wild.

### Natural variation of circadian traits and their potential role in adaptation to natural environments

The free-running period as determined in the laboratory can display high natural diversity across and within species, populations, and maternal families within populations (Table 1). In many cases, the period is relatively conserved within populations, although high variation has been observed within some populations and even within families. For example, period length within some wild populations of *Brassica rapa* periods can differ up to 4 hours (Lou *et al.*, 2011). In *Boechera stricta*, although some populations have extremely tight circadian periods with intra-population and intra-family variation of less than 30 minutes, some populations display up to ∼8 hours of intra-population variation and 5 hours of intra-family variation (Salmela *et al.*, 2016; McMinn *et al.*, 2022).

Correlations between this variation in period and environmental variables have been identified in a wide range of species, indicating that differences in period length help adapt plants to natural conditions. Positive correlations between latitude (day length) and free-running periods have been observed in wild accessions of *Arabidopsis thaliana*, *Mimulus guttatus*, and wild potato species (Table 1). In all cases, latitude variation explains less than 36% of the variation and in most cases the r-squared value was lower than 0.010, indicating that other factors are involved in the selection of particular circadian behaviours. It is also important to note that accession choice affects the detection of these correlations. For example, although an initial study observed a correlation between latitude and period in *Arabidopsis thaliana* (Michael *et al.*, 2003b), another study on a different set of accessions did not (Edwards *et al.*, 2005). Interestingly, a positive correlation between period and latitude has been observed in annual *Mimulus guttatus* populations but not perennial ones (Greenham *et al.*, 2017). In contrast, a negative correlation between period and latitude appears to be present in *Lemna aequinoctialis*, such that shorter periods are associated with higher latitudes, although it is important to note that only four populations were used in this study (Muranaka *et al.*, 2022).

Long circadian periods have been associated with high latitudes and long photoperiods during the growing season. It has been hypothesized that a delay in phase caused by a slower circadian clock could enable plants to use the extended light period found during long summer days. Positive correlations between period and growth have been found in multiple species within and across populations. Longer circadian periods have been associated with more biomass under long days in Arabidopsis recombinant inbred lines (Rubin *et al.*, 2017). *Boechera stricta* is a short-lived Rocky Mountain perennial that grows at high elevations and overwinters as a rosette. Within one mid-elevation (∼2572 m) population, the free-running period also positively correlates with more early vegetative growth and rosette size at flowering when grown under laboratory conditions simulating natural summer photoperiods and average temperature (Salmela *et al.*, 2016, 2018). Arabidopsis recombinant inbred lines display a negative correlation between photosystem II yield (Fv’/Fm’) and circadian period. However, it is important to note that for these photosynthesis measurements the plants were grown under short-day conditions, and this effect could be due to the lack of capacity of plants with long circadian periods to adapt to these short-day lengths. In accordance, the long-period plants had higher non-photochemical quenching even under lower light conditions (Yarkhunova *et al.*, 2016). Cultivated tomato is grown at higher latitudes and displays a longer circadian period than wild tomato species which originate from regions close to the equator (Müller *et al.*, 2016). The late phase caused by a cultivated tomato allele of *EID1* is linked to higher chlorophyll content under very long days (18 hours of light) but not under short days, suggesting that changes in photosynthesis rates could affect plant performance in a photoperiod specific manner. The circadian clock has been shown to modulate photosynthesis rates and most photosynthesis-related genes are under circadian control (De Barros Dantas *et al.*, 2023). However, further studies are needed to elucidate how circadian period control might modulate photosynthesis rates in a photoperiod dependent manner.

Significant correlations between period length and elevation have also been found. A negative correlation between period and elevation has been observed in *Arabidopsis thaliana* accessions (Edwards *et al.*, 2005) and *B. stricta* populations (McMinn *et al.*, 2022). Moreover, in *B. stricta* intra-population period range was smaller at higher elevation (McMinn *et al.*, 2022), indicating that variation of circadian traits might be of advantage to some populations but not in others. In Arabidopsis, the *ELF3* variant found in the Shahdara accession that causes a short period in constant light, a weaker shade avoidance response, and early flowering, is found in multiple other high elevation accessions (Jiménez-Gómez *et al.*, 2010; Coluccio *et al.*, 2011; Anwer *et al.*, 2014). This variant could be used as a model to investigate how clock-controlled traits potentially provide a fitness advantage under high elevations.

The free-running period of the circadian clock is temperature compensated, and is relatively stable when compared to other biochemical processes. Multiple clock genes are involved in temperature compensation of the Arabidopsis clock (Gil and Park, 2019; Maeda *et al.*, 2024). However, it is possible that temperature sensitivity might vary depending on the temperatures plants are exposed to in their natural habitats, in particular, because temperature under both stress and non-stress conditions influences circadian rhythms (Kolmos *et al.*, 2014; Gil and Park, 2019; Kidokoro *et al.*, 2021, 2023; Maeda *et al.*, 2024). Variation in temperature compensation of the free-running period has been observed in Arabidopsis (Edwards *et al.*, 2005; Kusakina *et al.*, 2014). A significant number of these Arabidopsis accessions display an acceleration of the clock at elevated temperatures. This phenomenon is similar to what has been observed among wild barley accessions; interestingly, fewer cultivated accessions exhibit this acceleration (Dakhiya *et al.*, 2017; Prusty *et al.*, 2021). In Arabidopsis, shortening of the period at higher temperatures has been associated with better growth at that elevated temperature (Kusakina *et al.*, 2014). In a similar manner, in barley, shorter periods at high temperatures are linked to increases in plant dry weight and a higher Rfd (a fluorescence parameter linked to plant vitality) and are associated with wild populations from arid regions (Dakhiya *et al.*, 2017; Lichtenthaler, 2021). Interestingly, the temperature-dependent plasticity of the period seems to be dependent on the photoperiod (Prusty *et al.*, 2021), indicating that multiple signals are integrated to achieve the optimal circadian period given the environmental condition.

Natural variation in the circadian rhythm robustness has also been detected in different plant species, often associated with responses to temperature. Weaker rhythms at elevated temperatures have been observed in many Arabidopsis accessions (Kusakina *et al.*, 2014). *Nothofagus pumilio*, a high-elevation (1000–1700 m) species, strongly loses rhythmicity at low elevation *in situ* conditions, although the related low-elevation species, *Nothofagus obliqua*, found at 680–900 m, is rhythmic at low elevations (Estravis-Barcala *et al.*, 2025). This effect is related to temperature sensitivity of the oscillations since laboratory experiments demonstrated that both species had rhythmic expression of clock-associated genes at 20 °C, but only the low elevation species maintained rhythmic expression at 30 °C and 34 °C. This temperature-based loss of rhythmicity in *N. pumilio* was correlated with a decrease in seedling survival and dry weight, while no such reduction in performance was observed in the more rhythmically robust *N. obliqua* (Estravis-Barcala *et al.*, 2025). Contrasting results were obtained for duckweeds. Within the genus *Wolffiella*, which originates from tropical climates, many species display arrhythmicity at higher temperatures; however, this loss of rhythmicity did not correspond to differences in growth at the higher temperatures (Isoda *et al.*, 2022). These different relationships between rhythm robustness and growth might be caused by the different growth and survival strategies between fast and slow growing plant species.

### Seasonal sensitivity and the circadian period

Correlations between circadian period variation and latitude have been linked to photoperiod sensing mechanisms regulating developmental transitions. Some of these transitions such as flowering in Arabidopsis are triggered when the day length is longer than a particular threshold, also known as the critical photoperiod (Blackman, 2017). In contrast, other responses, such as tuberization in potato, are activated when the days are shorter than the critical photoperiod (Abelenda *et al.*, 2011). Advances in the elucidation of the role of the clock on the photoperiod sensing mechanisms in the model plant Arabidopsis and other species are providing a framework to understand how variations in the period can affect photoperiod sensing in natural populations.

**Fig. 3.**
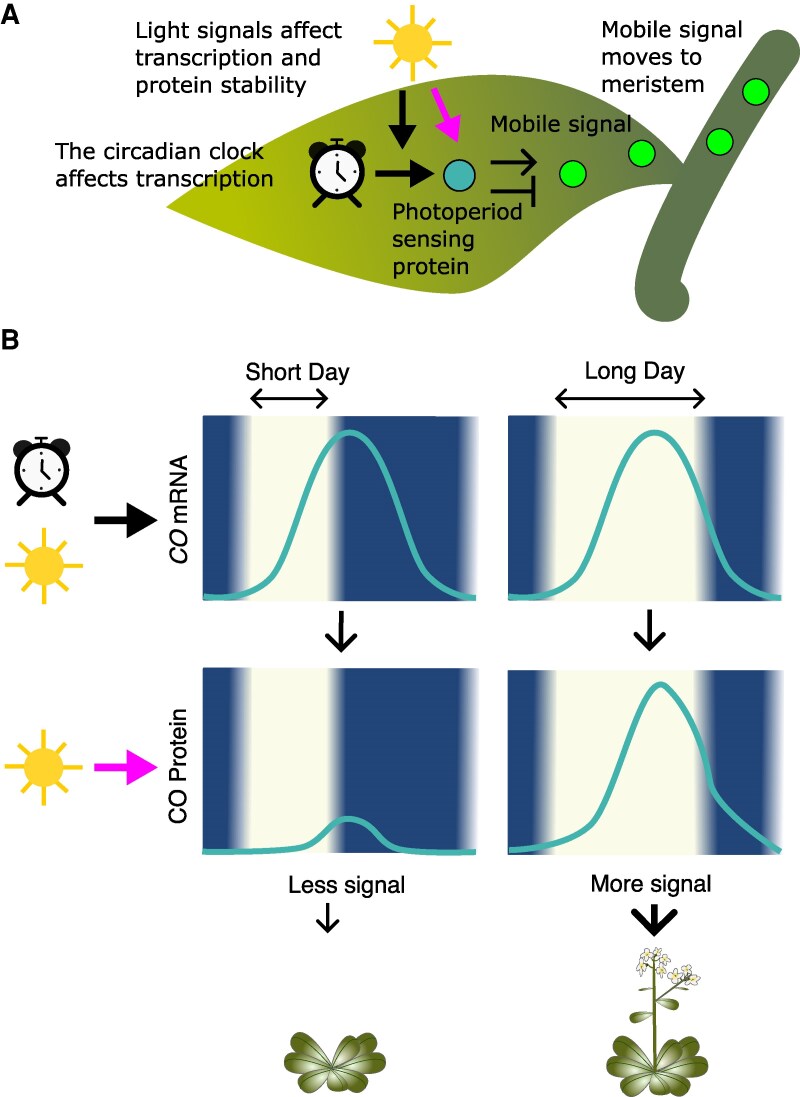
Simplified model for photoperiod sensing in plants. (A) The transcription of a photoperiod sensor is regulated by the circadian clock and light signals (black arrows). The protein stability of the sensor is regulated by light (magenta arrows). The sensor can act negatively (short-day responses) or positively (long-day responses) on the accumulation of a mobile signal, which moves to the meristem to trigger developmental responses. (B) Simplified model of long-day induction of flowering in *Arabidopsis*. The transcription of *CO* is controlled by the clock and light signals such that its mRNA peaks at the end of the light period. Light is needed for the stabilization and accumulation of CO protein, so that it only accumulates during long days enabling the production of the mobile signal FT (FLOWERING LOCUS T).

In these systems, the circadian clock determines the permissive time window leading to the light-dependent production of regulatory proteins (Imaizumi, 2025). These photoperiod-dependent regulators control the expression of mobile signals that mediate developmental transitions in a daylength dependent manner (Fig. 3A). For example, in the model plant Arabidopsis, clock-controlled *CONSTANTS* (*CO*) is expressed during the second half of the light period (Fig. 3B). The CO protein is degraded in the dark and only active during the day. Therefore, only if *CO* mRNA is present during the light period does CO protein accumulate (Fig. 3B). In Arabidopsis, flowering is a ‘long day’ response and CO, the light-sensitive protein, acts positively on the production of a long-distance signalling protein, FLOWERING LOCUS T (FT), which in turn induces flowering after moving to the meristem. In contrast, in the case of ‘short day’ responses, it appears that the light-regulated protein acts as a repressor of flowering (González-Delgado *et al.*, 2025a). For example, in domesticated rice, *Grain Number, Plant Height, and Heading Date7* (Os*Gdh7*) is induced in the light and encodes for a light stabilized protein that acts as a repressor of flowering (Itoh *et al.*, 2010; Zheng *et al.*, 2019). In the potato landrace *Solanum tuberosum* group *andigena*, an orthologue of the Arabidopsis *CO*, St*CO1*, also encodes for a light stabilized protein which acts negatively on tuberization (Abelenda *et al.*, 2016). A similar mechanism has been proposed to occur for tomato flowering time regulation (González-Delgado *et al.*, 2025a).

**Fig. 4.**
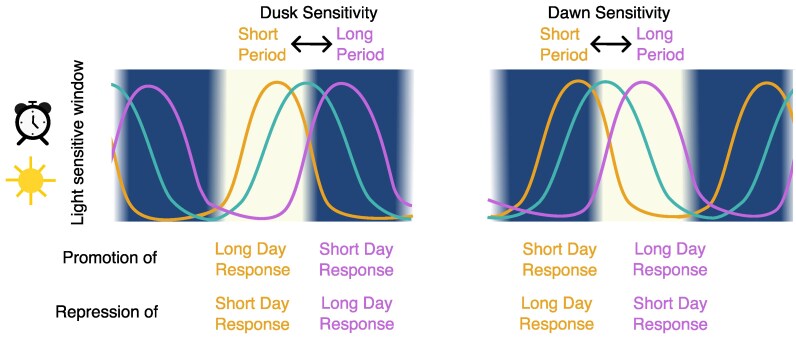
Model representing effect of period length on photoperiod responses. The circadian clock and light signals control the permissive window of expression of a photoperiod sensing gene. When that permissive window overlaps with the day, long-day responses are promoted and short-day responses are repressed. In contrast, when that permissive window overlaps with the night, long-day responses are repressed and short-day responses are induced. Therefore, the effect of the period length on this signal depends on whether the permissive window occurs around dusk (left panel) or dawn (right panel).

The timing of the light-sensitive window will influence the circadian effect on the photoperiod response (Fig. 4). For example, in Arabidopsis the light sensitive window for CO accumulation is in the evening (Valverde *et al.*, 2004). In contrast, in domesticated rice, the key sensitive period appears to be the morning (Itoh *et al.*, 2010; Zheng *et al.*, 2019). Theoretically, these differences will generate different responses to advances and delays of circadian timing. Thus, an advance in phase of an evening light-sensitive window will lead to an increase in a light-dependent response since a greater proportion of that window will coincide with the light period. However, a similar advance in phase will have a different effect on a system in which the light-sensitive window occurs in the morning, since an advance will lead to a larger overlap with the dark period (Fig. 4). If we also take into account that light signals appear to promote transitions in long-day plants and repress them in short days, this model could explain many of the observed relationships between the circadian period and photoperiod responses.

In Arabidopsis mutants, a short period is associated with early flowering since an early phase leads to an early peak of *CO* mRNA, i.e. more mRNA during the light period, enabling a higher accumulation of CO protein and therefore induction of flowering even under shorter days (Yanovsky and Kay, 2002; Valverde *et al.*, 2004). In natural Arabidopsis accessions, a long period and late phase have also been linked to a delay in flowering (Salmela and Weinig, 2019; Rees *et al.*, 2021). Arabidopsis photoperiod signalling pathways are complex and also include dawn signalling components (Song *et al.*, 2018; Lee *et al.*, 2023), therefore more studies are needed to evaluate the role of the circadian period on the phase of *CO* mRNA levels. A short period and/or early phase is also associated with early flowering in long-day crops such as barley (Helmsorig *et al.*, 2024) and wheat (Wittern *et al.*, 2023), indicating that their photoperiod sensing might be regulated using a similar principle.

Short circadian periods and early phases have been associated with early flowering in the short-day monocot *L. aequinoctialis. Lemna aequinoctialis* displays natural variation in the circadian period (Muranaka *et al.*, 2022). A shortening of the circadian period is associated with an early phase under diel conditions and a longer critical photoperiod that leads to flowering earlier in the season. Earlier flowering was associated with higher latitudes across the 72 strains tested. Interestingly, one population had a shorter period and earlier flowering time than expected based on latitude alone. This population mainly grows in rice paddy fields and its early flowering phenotype was associated with an earlier cessation of paddy field flooding at that location. These observations indicate that, at least in *L. aequinoctialis*, photoperiod sensing is the main driver for the natural variation of the circadian period by helping these plants adapt to different latitudes and other growth restricting environmental variables.

In *L. aequinoctialis*, the *FT* homologue La*FTh* acts as a flowering inducer (Muranaka *et al.*, 2022). It is expressed when its permissive time window, which is determined by the circadian clock and occurs around dawn, overlaps with the dark. A larger overlap between the dark period and the permissive window leads to higher La*FTh* expression and thus a stronger flowering time response. In genotypes with a long circadian period and thus a late phase of the permissive window of La*FTh* expression, this window is more likely to coincide with the morning light and cause an inhibition of La*FTh* expression. Therefore, these long-period plants require shorter days, i.e. longer nights, to flower. These results indicate that *L. aequinoctialis* has a dawn light sensitivity window (Fig. 4). More studies are needed to understand how the circadian clock and light signals regulate La*FTh* expression.

Growth cessation and bud set is strongly regulated by the photoperiod in poplar (Triozzi *et al.*, 2018). Bud set is initiated when the photoperiod is shorter than a critical daylength. Across natural accessions an early phase under diel conditions is associated with a shorter critical photoperiod and a delay in growth cessation at lower latitudes (Böhlenius *et al.*, 2006). It is unclear whether these natural accessions display differences in the circadian period; however, transgenic poplar with decreased expression of either Ptt*TOC1* or Ptt*LHY1* and Ptt*LHY2* led to an early phase under light/dark cycles, a short circadian period, and a delayed bud set (Ibáñez *et al.*, 2010). In contrast, a decrease in Ptt*PhyA* expression leads to a longer period and early growth cessation (Kozarewa *et al.*, 2010). It is still unknown whether circadian defects underlie the natural variation in diel phase and critical daylength in poplar. It is also unknown which protein mediates photoperiod sensing in these trees. Ptt*FT2*, an orthologue of Arabidopsis *FT*, promotes growth and inhibits growth cessation, and its expression is regulated by daylength (Böhlenius *et al.*, 2006). However, changes in Ptt*CO1* and Ptt*CO2* expression have only a weak effect on growth cessation (Böhlenius *et al.*, 2006; Hsu *et al.*, 2012). As the poplar photoperiod sensing protein remains to be discovered, the relationship between phase and critical day length, according to the model (Fig. 4), indicates that this gene might mediate a light-sensing window at the end of the day in a similar manner as in Arabidopsis (Fig. 3).

Within a *B. stricta* Rocky Mountain population, a significant genetic variation in circadian period was associated with variation in flowering time (Salmela *et al.*, 2018). A long period correlated with earlier flowering after vernalization under simulated summer solstice conditions. This earlier flowering was also correlated with a higher number of leaves and a larger rosette diameter at flowering. Studies on *B. stricta* indicate that, in the laboratory, photoperiod (12 hours versus 16 hours) does not strongly influence flowering after vernalization (Anderson *et al.*, 2011). These observations suggests that in this case the regulation of flowering by the clock might not be related to photoperiod sensing but maybe indirect via the regulation of vegetative growth.

Plants have evolved multiple strategies to regulate their developmental transitions in such a way as to thrive in diverse habitats (Blackman, 2017). Photoperiod sensing appears to be one of the main targets of how natural variation of the clock modulates flowering time. Although the architectures of the photoperiod-sensing mechanisms are similar across different plant species, differences in timing of expression and downstream regulators seem to enable changes in the speed of the clock to have different effects on the critical photoperiod (Figs 3 and 4). The identification and characterization of photoperiod sensing proteins and their expression patterns in species with different regulatory mechanisms are needed to confirm this hypothesis.

## Conclusion

Circadian traits display significant variation within and across plant populations. This variation is associated not only with differences in plant performance traits, such as growth, photosynthesis or flowering time, but also with different geographic locations indicating a role of the clock in local adaptation. The strong pleiotropic functions of circadian clock genes make it difficult to understand how a change such as the circadian period can affect multiple pathways in such a way that helps optimize plant survival in multiple species and environmental niches. Work on Arabidopsis and crop models has demonstrated that allelic variation of clock-associated genes can lead to a wide range of phenotypic variation that, in the case of crops, has been linked to the expansion of the cultivation range. However, we still know little about how trade-offs across different circadian-controlled processes interact during the natural selection process and the role of timing in most clock-controlled stress responses. The photoperiodic control of flowering time has been the most studied circadian output across a wide range of plant species. Based on these studies a model emerges that shows how similar circadian traits can lead to different responses and potentially minimize phenotypic trade-offs. In the case of photoperiodic control of flowering time two mechanisms seem to allow for different responses to similar changes in the circadian trait. One is based on changes of the timing of the clock-controlled signalling component that enables the sensing of light at the most appropriate time of day. The other is based on changes in downstream regulators that enable either inhibition or activation of the trait. It is possible that the increase in clock-associated gene copy number in higher plants enables fine tuning of timing, environmental sensing, and output control to optimize plant growth and development in a wide range of geographic niches.
